# Metal transport protein 8 in *Camellia sinensis* confers superior manganese tolerance when expressed in yeast and *Arabidopsis thaliana*

**DOI:** 10.1038/srep39915

**Published:** 2017-01-04

**Authors:** Qinghui Li, Yue Li, Xiayuan Wu, Lin Zhou, Xujun Zhu, Wanping Fang

**Affiliations:** 1 College of Horticulture, Nanjing Agricultural University, Nanjing 210095, P. R. China; 2 Botanical Gardens, Tohoku University, Aoba, Sendai 980-0862, Japan; 3 College of Biotechnology and Pharmaceutical Engineering, Nanjing Tech University, Nanjing 211800, P. R. China

## Abstract

Manganese (Mn) is an important micronutrient element required for plant growth and development, playing catalytic roles in enzymes, membranes and DNA replication. The tea plant (*Camellia sinensis*) is able to accumulate high concentration of Mn without showing signs of toxicity, but the molecular mechanisms underlying this remain largely unknown. In this study, the *C. sinensis* cultivar ‘LJCY’ had higher Mn tolerance than cultivar ‘YS’, because chlorophyll content reduction was lower under the high Mn treatment. Proteomic analysis of the leaves revealed that *C. sinensis* Metal Tolerance Protein 8 (CsMTP8) accumulated in response to Mn toxicity in cultivar ‘LJCY’. The gene encoding CsMTP8, designated as *CsMTP8* was also isolated, and its expression enhanced Mn tolerance in *Saccharomyces cerevisiae*. Similarly, the overexpression of *CsMTP8* in *Arabidopsis thaliana* increased plant tolerance and reduced Mn accumulation in plant tissues under excess Mn conditions. Subcellular localization analysis of green florescence fused protein indicated that CsMTP8 was localized to the plasma membranes. Taken together, the results suggest that CsMTP8 is a Mn-specific transporter, which is localized in the plasma membrane, and transports excess Mn out of plant cells. The results also suggest that it is needed for Mn tolerance in shoots.

Manganese (Mn) serves as a cofactor in essential plant processes, including secondary metabolism, lipid biosynthesis, photosynthesis, and oxidative stress^1^,^2^,^3^. Plants exhibit decreased growth and yield and are more susceptible to pathogens and damage at freezing temperatures under Mn deficient conditions. In contrast, Mn is also an element that is required in both plants and animals, but can be potentially toxic^4^. Although a number of plants exhibit Mn toxicity under excess Mn conditions, it can induce brown spots on mature leaves^5^, interveinal chlorosis, necrotic and dwarf young leaves^6^, which symptoms are generally observed on plants growing in acid soils. Higher plants have developed a set of strategies for Mn acquisition and transportation because Mn is required for plant development^7^.

Heavy metals are firstly combined by low molecular weight compounds, which are followed by separation into organelles or translocation to the extracellular space by specific transporters to avoid cellular damage in plant cells. Several gene transporter families have been identified as being involved in Mn transport in plants. These transporter families include NRAMP (natural resistance associated macrophage protein)^8^,^9^, CAX (cation exchanger)^10^,^11^,^12^, CCX (calcium cation exchangers)^13^, IRT (iron-regulated transporter)^14^,^15^, CDF (cation diffusion facilitator)^16^,^17^,^18^, P-type ATPases^19^ and VIT (vacuolar iron transporter)^20^. The CDF family, whose group members are related to metal transportation roles, is also known as the Metal Tolerance Proteins (MTPs) in plants^21^,^22^.

Previous phylogenetic analyses have divided the CDF family into three major sub-families. These have been named Zn-CDFs, Fe/Zn-CDFs, and Mn-CDFs based on their respective major metal substrate^16^. They serve as cation transporters of Zn, Mn, Fe and Cd^23^. Generally, six putative trans-membrane domains (TMDs) are found in many CDF transporters, as well as a histidine-rich region that exists either between TMDs IV and V, or within the N- or C-termini, where it exhibits N- and C-termini localization in the cytoplasm^24^,^25^. The plant MTPs are further classified into seven groups, and group naming follows the nomenclature of the MTP sequences from *Arabidopsis thaliana*^23^. Groups 1, 5, and 12 belong to Zn-CDF; groups 6 and 7, to Fe/Zn-CDF; and groups 8 and 9, to Mn-CDF^23^. To date, there has been more research on the Zn-CDF group than the other groups. Transporting excess Zn from the cytoplasm into vacuoles is regulated by AtMTP1, which controls cellular Zn homeostasis^26^,^27^. AtMTP3 has also been shown to have maintenance functions during Zn and/or Co sequestration within plant cell vacuoles^28^. Genes encoding MTPs that belong to the Zn-CDF group are involving in Zn tolerance and homeostasis, and are also present in other plant species, such as *Medicago truncatula (MtMTP1*)^29^, rice (*OsMTP1*)^30^, *Arabidopsis halleri (AhMTP1*)^31^, and *Thlaspi goesingense (TgMTP1*)^32^. In contrast to the Zn-CDF group, few studies on the functional analysis of plant Mn-CDFs are reported. The Mn-CDF group has two distinct subgroups termed Groups 8 and 9^23^. *Arabidopsis thaliana* contains four members in the Mn-CDF group, of which AtMTP8 is included in Group 8, whereas AtMTP9/10/11 are members of Group 9. MTP11 from *A. thaliana* was localized to the Golgi apparatus and AtMTP11 is involved in Mn tolerance by loading Mn into secretory vesicles that removes Mn from the cell via exocytosis^33^. However, another study localized AtMTP11 to the pre-vacuolar compartment, which indicated that compartmentalization was the tolerance mechanism^34^. In rice, there are five members in the Mn-CDF group^23^, and OsMTP8/8.1 and OsMTP9/11/11.1 are classified into Groups 8 and 9, respectively. Apart from the Mn-CDF members from *A. thaliana* and rice, ShMTP8 (Group 8), isolated from the Mn-tolerant legume *Stylosanthes hamata*, localizes to the tonoplast and confers Mn tolerance when ectopically expressed in *A. thaliana*^35^. A recent study revealed that *OsMTP8*.*1* encodes a tonoplast-localized Mn transporter that confers Mn tolerance through the sequestration of Mn into rice shoot cell vacuoles^17^, and OsMTP8.1 silencing lines show plant growth inhibition under excess Mn, which indicates that *OsMTP8*.*1* is involved in Mn tolerance by compartmentalizing it in rice vacuoles^17^. Migocka and his colleagues reported that the vacuolar membrane localized cucumber-MTP8, a Mn transporter, which helps maintain Mn homeostasis in root cells^18^. The same group also found that CsMTP9 is a plasma membrane H^+^-coupled Mn^2+^ and Cd^2+^ antiporter involved in the efflux of Mn and Cd from cucumber root cells by transporting both metals from the endodermis into vascular tissues^36^. Golgi localized Mn^2+^ transport proteins, MTP8.1 and MTP8.2, are involved in barley intracellular Mn homeostasis^37^. Nevertheless, plant MTP family members in non-model plants are still largely unknown.

Tea plant (*Camellia sinensis* (L.) O. Kuntze), an evergreen woody plant, is able to accumulate high concentrations of Mn (which more than 1000 mg.kg^−1^), without showing signs of toxicity^38^. Under Mn-deficient conditions, Mn fertilization increases fresh tea production, but under Mn-sufficient conditions, tea production decreases when Mn fertilizer is applied^39^. In this study, we used proteomics analysis and cDNA ends (RACE) to identify and characterize one novel gene, *CsMTP8*, which encodes Mn^2+^ transport proteins. *MTP8* transcript levels in the leaves showed contrasting responses towards different external concentrations of Mn, which suggested its protein was transcriptionally regulated by the plant Mn nutritional status. This study reports the functional characterization of an MTP8-like protein in *C. sinensis*, which probably confers Mn tolerance through Mn efflux from shoot cells.

## Results

### Characterization of Mn tolerance in *C. sinensis*

**Figure 1:**
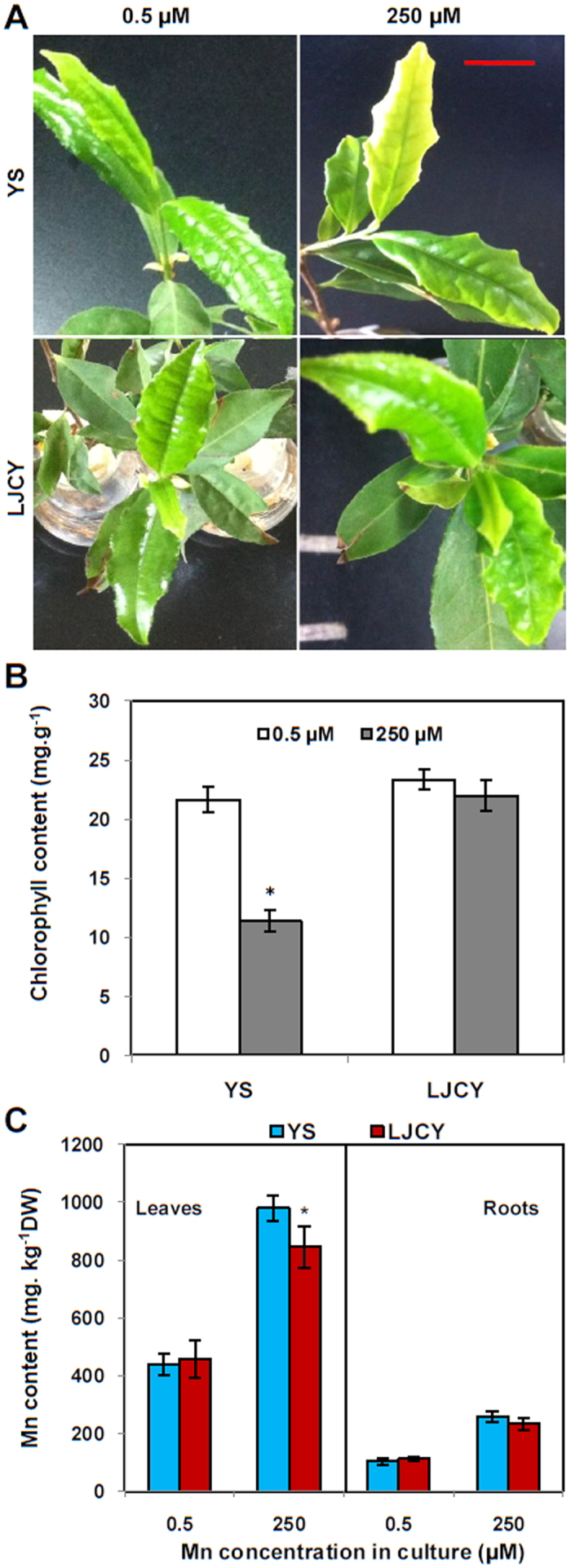
*C. sinensis* growth and Mn concentrations of Mn treatment. The *C. sinensis* seedlings were grown under normal conditions (0.5 μM MnSO_4_) for 1 month, and then treated with 0.5 (control) and 250 μM MnSO_4_ (excess) for 14 d. (**A**) Leaves of *C. sinensis* cultivars ‘YS’ and ‘LJCY’ after exposure to different Mn concentrations. (**B**) Chlorophyll contents of *C. sinensis* cultivars ‘YS’ and ‘LJCY’ exposed to different Mn concentrations. (**C**) Mn accumulation levels of *C. sinensis* ‘LJCY’ and ‘YS’ in leaves and roots after Mn treatment for 14 d. Data represent the mean ± SD (*n* = *4*). Asterisks indicate a significant difference at the P = 0.05 level. Bar = 1 cm.

The two *C. sinensis* cultivars (‘YS’ and ‘LJCY’) were exposed to 0.5 μM MnSO_4_ (control) and 250 μM MnSO_4_ (excess Mn) for 14 d. After the treatments, leaf chlorosis was observed in ‘YS’ when it was exposed to 250 μM MnSO_4_, but not in ‘LJCY’ (Fig. 1A). Leaf chlorophyll concentrations in ‘YS’ were 47.5% less under the excess Mn treatment than the Mn control solution, whereas the excess Mn treatment did not significantly affect leaf chlorophyll contents in ‘LJCY’ (Fig. 1B). Furthermore, the excess Mn treatment led to increased Mn concentrations in both the leaves and roots of *C. sinensis* ‘LJCY’ and ‘YS’ after 14 d. The Mn concentrations in ‘LJCY’ were significant less than ‘YS’ by 16.0% in the leaves, but not in the roots (Fig. 1C). The above results indicated that cultivar ‘LJCY’ had a greater Mn tolerance ability than ‘YS’, and that the leaves accumulate more Mn than the roots.

### Identification and the expression patterns of *CsMTP8* in *C. sinensis*

**Figure 2:**
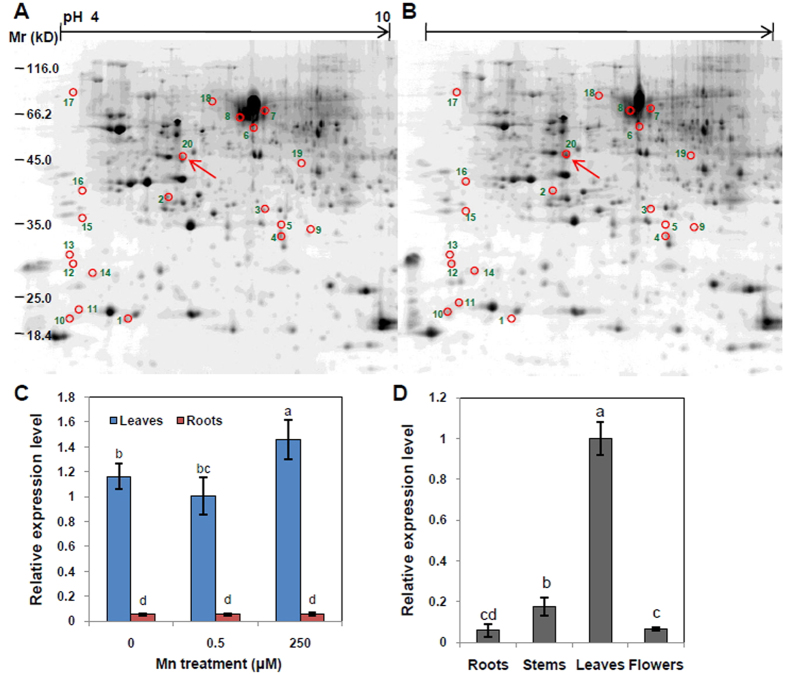
Identification and expression pattern of CsMTP8 in *C. sinensis*. 2-DE analysis of tea plant leaves grown under the control Mn (**A**, 0.5 μM) or excess Mn (**B**, 250 μM) treatments. (**C**) Quantitative real-time RT-PCR analysis of the *CsMTP8* transcript level in tea plant leaves and roots grown in media containing different Mn concentrations (0, 0.5, or 250 μM). Expressions are relative to the leaves exposed to 0.5 μM Mn. (**D**) Quantitative real-time RT-PCR analysis of *CsMTP8* expressions in the roots, stems, leaves, and buds. Data represent the mean ± SD (*n* = *4*). Different letters indicate a significant difference at the P = 0.05 level.

In order to identify differentially expressed proteins regulated by excess Mn in *C. sinensis* cultivar ‘LJCY’, the protein profiles of ‘LJCY’ leaves were analyzed using two-dimensional electrophoresis (2-DE) combined with matrix-assisted laser desorption ionization (MALDI)-time of flight (TOF)/TOF mass spectrometry (MS) analysis. More than 700 protein spots were detected by 2-DE analysis, and 23 protein spots exhibited differential expression between the control and excess Mn treatments (Fig. 2A,B). Furthermore, 20 protein spots (9 down-regulated and 11 up-regulated) were further identified by MALDI-TOF MS analysis and NCBI database search (Table S1). Subsequently, we analyzed one protein spot that was up-regulated more than 3-fold under the excess Mn treatment and named it *CsMTP8* (E20 in Fig. 2A,B and Table S1).

A 423 bp partial fragment of complementary DNA from *CsMTP8* was amplified using the conserved MTP8 motifs found in other plants. 5-RACE and 3-RACE were performed, based on the partial cDNA sequence of *CsMTP8*, to clone full length *CsMTP8* cDNA, which was 1, 215 bp in length and encoded a protein with a predicated molecular mass of 44.5 kDa. The *CsMTP8* transcript levels increased 1.5-fold in ‘LJCY’ leaves in response to 250 μM Mn compared to plants grown under the 0.5 μM Mn control conditions, and were slightly higher after the 0 μM Mn treatment. The *CsMTP8* transcript levels in tea plant roots grown under the three Mn treatments (0, 0.5, and 250 μM) showed little change. *CsMTP8* was mainly expressed in the leaves under all conditions, and excess Mn application resulted in *CsMTP8* transcription levels that were more than 17-fold higher than in the roots (Fig. 2C). The tissue expression patterns and responses were determined using quantitative real-time RT-PCR analysis. The *CsMTP8* levels in the roots, stems, leaves, and flowers showed that it is predominantly expressed in the leaves, and that the expression levels in other tissues were low, which suggested that *CsMTP8* is present in many different *C. sinensis* tissues, but the expression level varies depending on the tissue (Fig. 2D).

### *CsMTP8* sequence analysis

**Figure 3:**
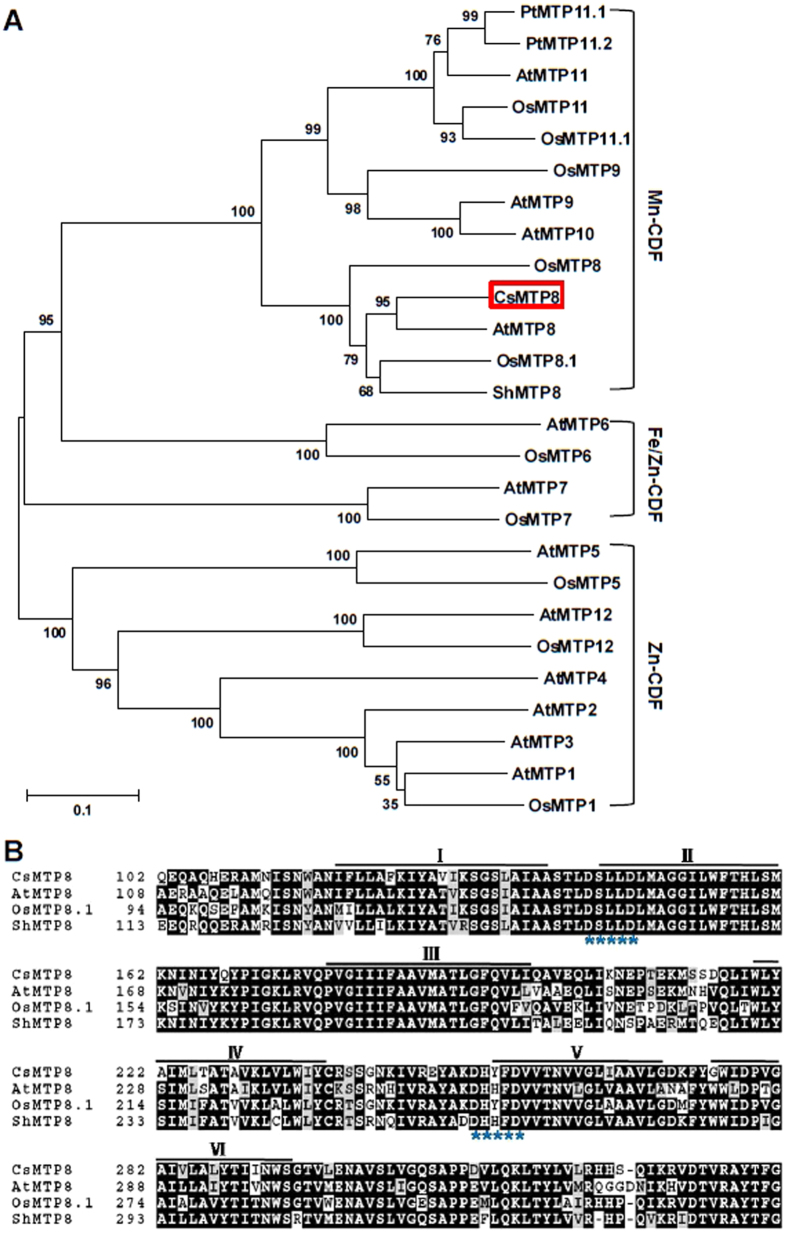
Sequence analysis. (**A**) Phylogenetic comparison between CsMTP8 and other plant MTPs. The analysis was performed using the neighbor-joining method with 1000 bootstraps by MEGA6 software. (**B**) Clustal X alignment of the MTPs from rice (OsMTP8.1), *Stylosanthes hamata* (ShMTP8), and *Arabidopsis thaliana* (AtMTP8). The transmembrane domains of CsMTP8 predicted by the HMMTOP program are shown as lines above the sequence. Asterisks indicate the conserved sequence (DxxxD) in members of the Mn-CDF group.

All MTPs were annotated based on the results of BLASTP searches against GenBank protein data sets using CsMTP8 and on the output of the phylogenetic analysis including MTPs from monocots and eudicots. The phylogenetic analysis results and the substrate specificities of certain characterized transporters showed that the CDF family members could be divided into three major subfamilies. These were Zn-CDF, Fe/Zn-CDF, and Mn-CDF (Fig. 3A). The *CsMTP8* sequence contains 405 amino acid residues, and it is predicted to contain six transmembrane domains (TMDs), and two conserve active sequences (DxxxD) for MTP8-like transporters. These are DSLLD within the putative TMDII, and DHYFD within the cytosolic loop preceding TMDV. The residues are present in OsMTP8.1 and in the Mn transporters AtMTP8, OsMTP8.1, and ShMTP8 (Fig. 3B).

### Effect of *CsMTP8* expression on Mn tolerance

**Figure 4:**
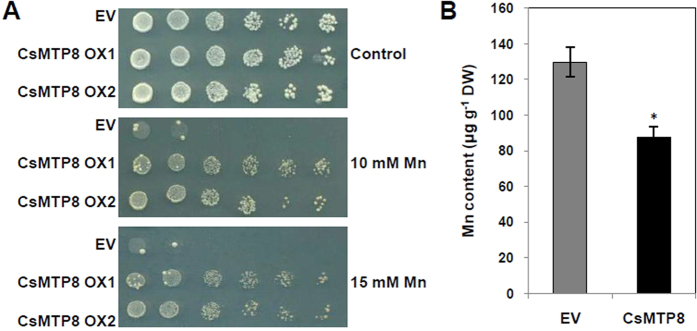
Effect of *CsMTP8* expression on Mn tolerance and Mn accumulation in *Saccharomyces cerevisiae*. (**A**) The yeast mutants INVSC1 carrying the pYES2 empty vector or pYES2-CsMTP8 were used. Yeast cells with optical densities of 2.0 at 600 nm (OD_600_) and samples taken after six gradient 1:10 dilutions were spotted onto synthetic complete medium containing 10 mM, and 15 mM MnSO_4_. They were left to culture for 48 h at 30 °C. EV indicates yeast cells transformed with the empty vector pYES2. OX1 and OX2 are two clones showing *CsMTP8* overexpression. (**B**) Mn accumulation in wild-type strain transformed with empty vector pYES2 or pYES2-CsMTP8. Following cultured in liquid SC-U/Gal medium supplemented with 5 mM MnSO_4_ at an initial OD_600_ = 0.1 for 24 h. Asterisks indicate a significant difference at the P = 0.05 level compared to EV.

CsMTP8 was functionally characterized by first determining whether its expression affected INSVC1 sensitivity to Mn. INSVC1 cultures carrying either the pYES2 empty vector or pYES2-CsMTP8 grew similarly in media containing the control, 10, or 15 mM Mn. *CsMTP8*-overexpressing yeast cells were subjected to excess Mn to test whether *CsMTP8* was involved in tolerating Mn toxicity. Figure 4A indicates that the yeast cells harboring *CsMTP8* showed attenuated sensitivity to excess Mn compared to those harboring the pYES2 empty vector control, which suggests that the pYES2-CsMTP8 vector improved manganese tolerance to Mn at 10 mM and 15 mM MnSO_4_ (Fig. 4A). Furthermore, the Mn accumulation levels were compared in wild-type strains which expressed *CsMTP8* and empty vector for investigating the metal transport activity. When the strains were exposed to 5 mM Mn^2+^, cells expressing *CsMTP8* accumulated significantly less Mn than the empty vector control after 24 h (Fig. 4B), indicating that CsMTP8 detoxifies cells by an efflux of Mn^2+^ to the external medium.

### Subcellular localization of *CsMTP8* protein in onion epidermal cells

**Figure 5:**
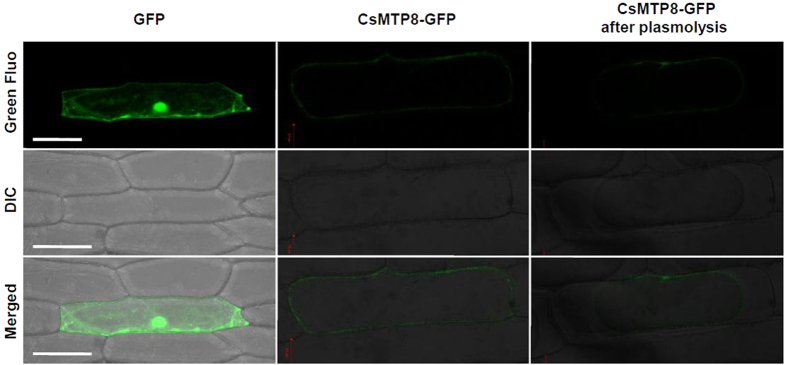
Subcellular localization of CsMTP8 protein in onion epidermal cells. Onion epidermal cells, including before and after plasmolysis, were transformed with GFP and CsMTP8-GFP fusion protein. Images were obtained in a dark field to detect green fluorescence, in bright light to observe the morphological characteristics of the cells, and in combination. Bars = 100 μm.

A CsMTP8: GFP fusion protein was transiently expressed in onion epidermal cells so that the biological functions and localization of *C. sinensis* protein could be determined. Fluorescent cells transfected with GFP alone were detected in the nucleus and cytoplasm. In contrast, CsMTP8-GFP fusion protein fluorescence was observed at the cell periphery, but not in the nucleus, especially after plasmolysis, which suggested that CsMTP8 localized to the plasma membrane (Fig. 5). Furthermore, the WoLF PSORT protein subcellular localization prediction software predicted that CsMTP8 was a membrane localization protein^40^.

### Effect of *CsMTP8* overexpression in *Arabidopsis thaliana* on Mn tolerance

**Figure 6:**
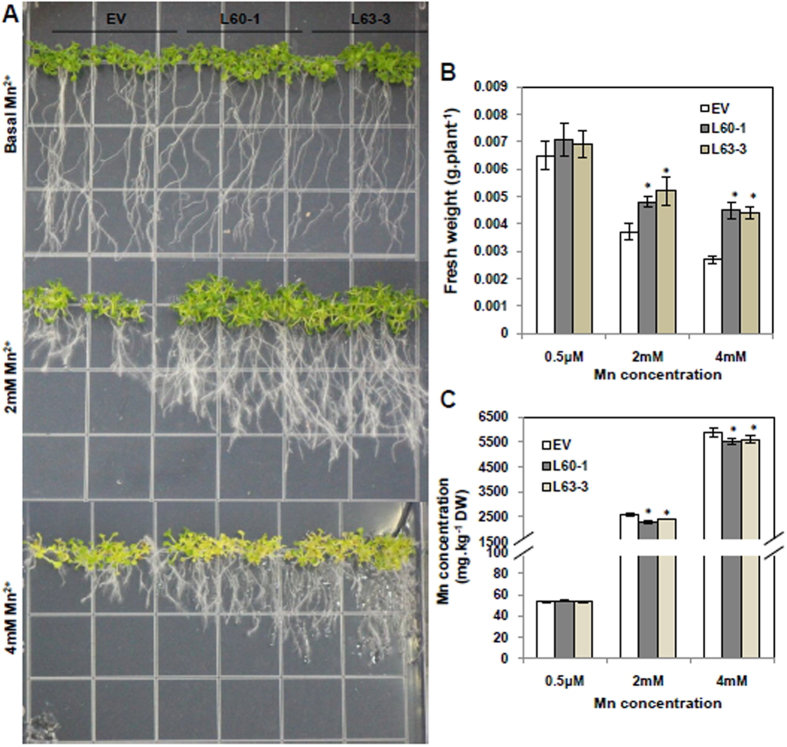
Effect of *CsMTP8* overexpression in *Arabidopsis thaliana* on Mn tolerance. (**A**) Growth of *A. thaliana* under the different Mn treatments for 14 days. (**B**) Fresh weights of the *A. thaliana* plants. (**C**) Mn concentrations in *A. thaliana* plants. 5-day-old seedlings were transferred into 1/2 MS supplemented with 2 mM or 4 mM for 14 days. EV indicates the wild-type *A. thaliana* Columbia ecotype that was transformed with the empty vector. L60-1, L63-3 are two transgenic lines that expressed *CsMTP8*. Asterisks indicate a significant difference at the P = 0.05 level compared to EV.

*CsMTP8* was overexpressed in *A. thaliana* to investigate whether elevated CsMTP8 expression led to increased Mn accumulation and the enhanced Mn tolerance in the plants. Two independent homozygous transgenic lines, L60-1 and L63-3 (Figure S1), were selected from several transgenic lines to determine the effect of excess Mn on Mn accumulation and plant growth. The expression of the *C. sinensis* gene in *Arabidopsis* led to increased expression of the *CsMTP8* gene (Fig. 6A). Figure 6A shows that there was no difference in *Arabidopsis* growth between the *CsMTP8* overexpression L60-1 and L63-3 transgenic lines, and the empty vector under the control MnSO_4_ conditions. However, the seedlings transformed with the empty vector grew poorly under toxic Mn conditions (2 mM and 4 mM MnSO_4_). As the Mn levels rose, the fresh weight decreased more severely in the empty vector than in the *CsMTP8* transgenic line L63-3, with the final weight being 40.5% and 62.9% less for the empty vector than that for *CsMTP8* transgenic lines grown at 2 mM and 4 mM MnSO_4_, respectively (Fig. 6A,B). Furthermore, Mn concentrations in the *CsMTP8* overexpression transgenic lines were 11.5% and 6.5% less than those in the empty vector grown at 2 mM and 4 mM MnSO_4_, respectively (Fig. 6C). The two *CsMTP8* overexpressed and empty vector *A. thaliana* plants were also grew in other metals stress conditions. However, the transgenic lines of L60-1, L63-3 and the empty vector plants showed similar phenotype and reduced freshweight in toxic Co or Zn concentrations (Figure S2), which indicated that CsMTP8 is a Mn specific transporter. These results suggest that the over-expression of *CsMTP8* in *Arabidopsis* plants confers Mn tolerance by reducing Mn accumulation in plants, and that this may occur through efflux of excess Mn.

## Discussion

Manganese toxicity is one of the critical constraints limiting plant growth, especially in acid soils. Excess Mn can induce brown spots on mature leaves^5^, interveinal chlorosis, and necrotic and deformed young leaves^6^. Some plants that adapt well to acid soils may have specific strategies that give them a superior ability to tolerate Mn toxicity^35^,^41^. After 14 d of treatment, leaf chlorosis was observed in ‘YS’ when cultured in 250 μM MnSO_4_, but not in ‘LJCY’ (Fig. 1A). This suggested that *C. sinensis* ‘LJCY’ has superior Mn tolerance. In addition, Fig. 1B shows that Mn tolerance in *C. sinensis* ‘LJCY’ was due to it being able to avoid significant reductions in leaf chlorophyll contents when cultured at 250 μM Mn. In contrast, leaf chlorophyll concentrations in ‘YS’ were 47.5% less under excess Mn conditions compared to the Mn control treatment.

After 14 d of Mn treatment, the Mn concentrations of ‘LJCY’ and ‘YS’ increased both in the leaves and roots when the plants were exposed to excess Mn (Fig. 1C). However, the Mn concentrations in ‘LJCY’ were significant less than ‘YS’ by 16.0% in the leaves, but not in the roots (Fig. 1C). At 250 μM Mn, moisture adsorption also led to metal ion adsorption and the secretion of organic acids that chelated heavy metal ions. Manganese toxicity would lead to more heavy metal ions accumulating in the tea plant^42^. Isolation of a heavy metal is an effective way to reduce heavy metal in the cytoplasm^43^. In our study, the Mn contents in the leaves increased more than in the roots under Mn stress, and the *C. sinensis* ‘LJCY’ leaf Mn concentrations were about four-fold greater than in the roots at 250 μM Mn (Fig. 1C).

A proteomics analysis was undertaken using ‘LJCY’ leaves in order to elucidate the molecular mechanism in *C. sinensis* cultivar ‘LJCY’ that leads to higher Mn tolerance. High Mn levels up-regulated *CsMTP8* protein (Fig. 2A,B), and its expression level in the leaves was higher than in the roots under excess Mn conditions (Fig. 2C,D). These results suggest that *CsMTP*8 expression in *C. sinensis* leaves is up-regulated under excess Mn treatment at the transcription level, and that *CsMTP8* expression is specifically increased by Mn toxicity.

Recently, several MTPs from different plant species (*A. thaliana, O. sativa, M. truncatula, S. hamata*, and *C. sativus*) were characterized and reported. The phylogenetic and functional analyses of the MTPs in the plant species showed that the newly identified *C. sinensis* MTP could be classified into one of the three main subfamilies: Zn-CDF, Zn/Fe-CDF, or Mn-CDF. The functions of *MTP* genes can be generally predicted based on phylogenetic relationship analysis. According to the domain analysis^23^, *CsMTP8* belongs to the Mn-CDFs. Manganese tolerance that involves the MTP8-mediated sequestration of Mn out of the cells could determine the constitutive traits for continuous Mn detoxification in Mn hyperaccumulators. It has already been suggested that the *MTP8* gene expressions are related to the primary radiation of plants that moved onto land and that this had a significant effect on Mn homeostasis in plants^23^. As previously reported, OsMTP8.1 has a critical role in Mn detoxification by sequestering Mn into shoot vacuoles^17^. Furthermore, cucumber MTP8 is localized in the vacuolar membrane and participates in root cell Mn homeostasis^18^.

The function of *CsMTP8* in yeast and *Arabidopsis* expressing the tea plant gene was studied in order to investigate how MTP8 proteins function in tea plants with moderate or high sensitivity to Mn. The full-length cDNA contained a signature sequence that was specific for the CDF family and the short motifs of five amino acids that are only found in the Mn-CDF subgroup. These were DSLLD within the putative TMDII and DHYFD within the cytosolic loop preceding TMDV^16^ (Fig. 3). The specific Mn transporters: AtMTP8, OsMTP8.1, ShMTP8, and CsMTP8 (cucumber), also exhibit the motifs that are characteristic of the Mn-CDF subfamily. Furthermore, the overexpressed lines of L60-1, L63-3 exhibited similar phenotype and reduced freshweight with empty vector plants in toxic Co or Zn cultivation levels (Figure S2). The above results suggested that *CsMTP8* is a putative Mn transporter in tea plants.

The heterologous expression of *CsMTP8* in yeast cells resulted in enhanced tolerance to Mn (Fig. 4A), and CsMTP8 detoxifies cells by an efflux of Mn^2+^ to the external manner (Fig. 4B). Subsequent CsMTP8 was localized to the plasma membrane (Fig. 5), a localization that was further verified by plasmolysis, which suggested that *CsMTP8* is involved in transporting Mn^2+^ under excessive Mn conditions. Although all the *A. thaliana* fresh weights decreased when Mn increased 4000-fold and 8000-fold, the fresh weight decreased more severely in the empty vector than in *CsMTP8* overexpression transgenic lines, and Mn concentrations in *CsMTP8* overexpression plants were less than in the empty vector (Fig. 6). This suggests that *CsMTP8* overexpression confers superior Mn tolerance in *A. thaliana* plants, and that the tolerance is partly realized through reduced Mn accumulation in the plants. The results also suggest that *C. sinensis* protein contributes to efflux of excess Mn in plant cells^18^.

In conclusion, our data suggest that *CsMTP8* from *C. sinensis* is a functional homologue of MTP8. Furthermore, the expression of *CsMTP8* in tea plant leaves is up-regulated by excess Mn. It is reasonable to conclude that CsMTP8 localizes to the plasma membrane; overexpression improves manganese tolerance in yeast and *A. thaliana*; that is plays a special role in Mn homeostasis, presumably via excess Mn efflux from the leaf cells; and that these activities are regulated by *CsMTP8* accumulation at the transcriptional level.

## Methods

### Plant materials and treatments

One-year-old tea plants of cultivars ‘Longjingchangye’ and ‘Yingshuang’ (abbreviated as ‘LJCY’ and ‘YS’ respectively), were transplanted from the botanic garden at Nanjing Agricultural University, China. The tea plants were grown under a 12-h light (28 °C)/12-h dark (22 °C) photoperiod regime during the first cultivation stage. Following this, the *C. sinensis* plants were grown for 1 month in a nutrient solution described by Wan *et al*.^44^, and then treated with 0.5 (control) or 250 μM MnSO_4_ (excess exposure) for 14 d. The leaves and roots were separately harvested, washed with deionized water, weighed, and stored at −80 °C for further analysis.

### Protein identification using 2-DE and MALDI-TOF/TOF MS analysis

About 1.0 g of fresh leaf was ground to a powder in liquid nitrogen. Then 10 mL of cold acetone (containing 10% TCA) was added as described by Zhou *et al*.^45^. The pellet was resuspended in lysis buffer (7 M urea, 2 M thiourea, 4% CHAPS, 0.2% (w/v) Bio-Lyte, 65 mM dithiothreitol) and vortexed for 45 min at 25 °C. After centrifuging the sample twice (14,000 rpm, 30 min at 4 °C), the protein was separated from supernatant, the protein concentration was measured with bovine serum albumin as a standard, and then analyzed using the Coomassie Brilliant Blue G-250 method^46^.

The proteins were separated using isoelectric focusing (IEF) based on the isoelectric point (pI) of proteins using a PROTEAN IEF System (Bio-Rad, Hercules, CA, USA), which utilized pH 4–10 immobilized protein gradient strips (17 cm). A total of 2.2 mg protein that had been dissolved in 500 μL rehydration buffer for each strip were rehydrated by loading the samples into the channels of the IEF tray and actively rehydrating the strips at 20 °C for 12 h. Then isoelectric focusing was performed at 20 °C, 60 kVh, and with a maximum current setting of 50 μA for each strip. The immobilized protein gradient strips were equilibrated according to Zhou *et al*.^45^ during the first and second equilibration steps after IEF. The methods used for the second dimension sodium dodecyl sulfate-polyacrylamide gel electrophoresis and gel staining were the same as those described by Zhou *et al*.^45^. Only those spots whose quantity had changed more than two-fold in each of three replicated experiments were collected.

The proteins that had been differentially expressed were destained and digested using the improved in-gel digestion method described by Katayama^47^. The spots excised from the above gel were analyzed using an ABI 4800 MALDI-TOF/TOF Plus mass spectrometer (Applied Biosystems, Foster City, CA, USA). The mass spectra (m/z range: 800–3500 Da) were processed and the parameters for the peak detection algorithm were used. Our mass spectrometer data were searched against the NCBI protein sequence database by an in-house MASCOT (version 2.1; Matrix Science, London, UK) search engine. The search parameters were the same as Zhou *et al*.^45^. The BLAST method was used to obtain more information about the above proteins.

### Isolation and characterization of *CsMTP8*

Total RNA was extracted from ‘LJCY’ leaves under the excess Mn condition (250 μM MnSO_4_) using the Trizol reagent (Invitrogen, Carlsbad, CA, USA). First strand cDNA was synthesized from 2 μg *DNase* I treated RNA using M-MLV reverse transcriptase (Promega, Madison, WI, USA). The primers used to clone *CsMTP8* were designed using the conserved *MTP8* motif sequences from *Arabidopsis thaliana* (NM_115668), *Stylosanthes hamata* (AY181256), *Oryza sativa* (Os03g0226400), and *Ricinus communis* (XM_002524216). A 423 bp fragment was amplified using the primers CsMTP8-DEG-F and CsMTP8-DEG-R (Table S2). The PCR product was cloned into a pMD18-T vector (Takara, Shiga, Japan) and sequenced. Then the full length cDNA for *CsMTP8* was cloned through rapid amplification of cDNA ends (RACE) according to manufacturer protocols (Clontech, Palo Alto, CA, USA).

### Subcellular localization of CsMTP8

The *CsMTP8* open reading frame (ORF) without a stop codon was amplified by PCR using *CsMTP8*-166-F and *CsMTP8*-166-R (Table S2) as primers. The PCR product was digested with *XbaI* and *KpnI*, and then cloned into a pJIT166-GFP vector. Subsequently, particle bombardment (PDS-1000/He biolistic particle delivery system, Bio-Rad) was used to transiently express the GFP fusion proteins in onion epidermal cells^48^. After incubation at 25 °C for 16 h in the dark, the epidermal layers were peeled off and observed using a fluorescence microscope (LSCM, TCS SP2, Leica, Heidelberg, Germany).

### Quantitative Real-Time PCR analysis

Real time RT-PCR was performed using the StepOne real time PCR system (Applied Biosystems) and SYBR Premix *Ex Taq* (TaKaRa). The primers were designed by Primer Express Software Version 3.0 (Applied Biosystems). *CsMTP8*-RT-F and *CsMTP8*-RT-R were used for the two-step real time RT-PCR that was performed using the following program: one cycle of 94 °C for 10 min, 40 cycles of 94 °C for 15 s, and 60 °C for 60 s. *Csβ-actin* was used as an internal control in this study. The amount of cDNA was calculated relative to the signals for a standard dilution of the respective PCR products using StepOne^TM^ v2.1 (Applied Biosystems).

### Overexpressing *CsMTP8* in *Arabidopsis* and functional analysis

The coding region for *CsMTP8* was amplified and digested with *Bam*HІ and *Sac*І, and subsequently recombined into the pGFP166 vector with a modified cauliflower mosaic virus (CaMV) 35 S promoter. The *CsMTP8* gene was introduced into wild-type (WT) *Arabidopsis* (Columbia ecotype) using the floral dip method^49^. The positive transformant screening process involved cultivating the transformants in an MS medium containing 50 mg L^−1^ hygromycin. The integration of the transgene in *Arabidopsis* was confirmed by reverse transcriptase polymerase chain reaction (RT-PCR) analysis. The expression of the *CsMTP8* gene in positive transformants was determined by quantitative RT-PCR.

The Mn contents in transgenic *Arabidopsis* plants were measured by germinating wild-type and transgenic seeds on modified Murashige and Skoog (MS) solid medium following sterilization. After 7 d cultivation in an incubator, healthy seedlings were selected and transplanted onto MS solid medium supplied with 0.1, 2, or 4 mM MnSO_4_. After another 7 d, the seedlings were harvested to determine the fresh weights and Mn concentrations as described above.

### Yeast transformation and Mn tolerance analysis

The *CsMTP8* ORF was amplified by PCR using the primers CsMTP8-pYES2-F and CsMTP8-pYES2-R (Table S2). The pYES2 and the overexpressing CsMTP8 vectors, which were digested using BamHI and EcoRI, were introduced into the yeast strain INVSC1 (Invitrogen) according to manufacturer protocols. The yeast transformants (three CsMTP8-overexprssed clones and one empty vector clone) were first cultured in liquid synthetic complete-uracil medium until the OD_600_ value reached 0.6, and then the pre-cultured yeast cells were adjusted to an OD_600_ value of 0.2 in the same medium used for Mn tolerance analysis. Subsequently, yeast cells in each of the dilutions (optical densities at OD_600_ were 0.2, 0.02, 0.002, and 0.0002) were spotted onto induction plates containing 2% (w/v) Gal, 1% (w/v) raffinose, 0.67% (w/v) yeast nitrogen base without amino acids, 0.1% (w/v) uracil dropout mix, and 2% (w/v) agar, which were supplemented with 0, 10, and 15 mM MnSO_4_. The plates were cultured at pH 5.0, for 2 or 4 d at 30 °C.

To determine Mn accumulation in yeast cells, the wild type strain transformed by the pYES2 empty vector or pYES2-CsMTP8 was cultured in liquid SC-U/Gal medium supplemented with 5 mM MnSO_4_ at an initial OD_600_ = 0.1 for 24 h. The cells were washed by 10 mM cooled EDTA (pH 5.0) and then dried at 70 °C for 12 h. The Mn contents of the dried samples were measured as described below.

### Chlorophyll content determination

The chlorophyll in the leaves of *C. sinensis* plants under the excess and control Mn treatments was extracted with 80% acetone after they had been ground with a mortar and pestle. The samples were then centrifuged for 10 min at 10,000 rpm and 4 °C. The chlorophyll content was measured according Lichtenthaler^50^ at 664 nm and 647 nm absorbance.

### Determination of the Mn concentration

*C. sinensis* plant leaves and roots were collected, dried, and then digested with concentrated HNO_3_ (60%) at 140 °C. The Mn concentrations of the digested samples were determined using atomic absorption spectrometry (AA-6800, Shimadzu, Kyoto, Japan).

## Additional Information

**How to cite this article**: Li, Q. *et al*. Metal transport protein 8 in *Camellia sinensis* confers superior manganese tolerance when expressed in yeast and *Arabidopsis thaliana. Sci. Rep.* **7**, 39915; doi: 10.1038/srep39915 (2017).

**Publisher's note:** Springer Nature remains neutral with regard to jurisdictional claims in published maps and institutional affiliations.

## Supplementary Material

Supplementary Information
