# Supplementary material for: Metal transport protein 8 in Camellia sinensis confers superior manganese tolerance when expressed in yeast and Arabidopsis thaliana
Source: Sci Rep. 2017 Jan 4;7:39915. doi: 10.1038/srep39915 (PMC5209735; doi:10.1038/srep39915)
Supplement: Supplementary Information [file srep39915-s1.doc]

**Title:** Metal transport protein 8 in *Camellia sinensis* confers superior manganese tolerance when expressed in yeast and *Arabidopsis thaliana*

**Authors:** Qinghui Li^1^, Yue Li^2^, Xiayuan Wu^3^, Lin Zhou^1^, Xujun Zhu^1,*^ & Wanping Fang^1,*^

**Affiliation:**

1. College of Horticulture, Nanjing Agricultural University, Nanjing 210095, P. R. China

2. Botanical Gardens, Tohoku University, Aoba, Sendai 980-0862, Japan

3. College of Biotechnology and Pharmaceutical Engineering, Nanjing Tech University, Nanjing 211800, P. R. China

Table S1. The identification of differentially accumulation protein spots between adequate and excess Mn levels.

| Spot No. | Protein name | Accession No.^a^ | Species | Theoretical | | Experimental | | Score^b^ | %cov (matching peptides)^c^ | Ratio^d^ |
| --- | --- | --- | --- | --- | --- | --- | --- | --- | --- | --- |
|  |  |  |  | *M*_r_  (kDa) | pI | *M*_r_ (kDa) | pI |  |  |  |
| E1 | Hypothetical protein | WP_039311068 | *Paenibacillus sp. IHB B 3415* | 28.98 | 7.75 | 20.36 | 5.05 | 597 | 27%(4) | 2.258 |
| E2 | PREDICTED: uncharacterized protein At5g02240-like | XP_010262541.1 | *Nelumbo nucifera* | 32.29 | 6.27 | 38.74 | 5.85 | 828 | 41%(6) | 2.282 |
| E3 | Triosephosphate isomerase family protein | XP_002316287.2 | *Populus trichocarpa* | 30.37 | 5.15 | 36.52 | 7.55 | 416 | 25%(4) | 2.102 |
| E4 | Germin-like protein | AEN02469.1 | *Camellia sinensis* | 30.82 | 7.73 | 32.05 | 7.93 | 472 | 19%(3) | 2.072 |
| E5 | PREDICTED: glutathione S-transferase-like | XP_011079702.1 | *Sesamum indicum* | 34.22 | 5.89 | 33.78 | 7.93 | 339 | 19%(4) | 4.046 |
| E6 | Ribulose-1,5-bisphosphate carboxylase/oxygenase large subunit, partial | ABB03930.1 | *Serpocaulon vacillans* | 9.92 | 9.66 | 56.10 | 7.41 | 210 | 49%(4) | 2.969 |
| E7 | Ribulose-1,5-bisphosphate carboxylase/oxygenase large subunit, partial | ABB03930.1 | *Serpocaulon vacillans* | 9.92 | 9.66 | 59.96 | 7.69 | 219 | 49%(4) | 2.126 |
| E8 | Hypothetical protein VIGAN_06209900 | BAT90812.1 | *Vigna angularis var. angularis* | 31.86 | 6.90 | 62.46 | 7.10 | 288 | 9%(2) | 5.623 |
| E9 | Germin-like protein | AEN02469.1 | *Camellia sinensis* | 30.82 | 7.73 | 32.700 | 8.43 | 294 | 18%(3) | 11.73 |
| E10 | PREDICTED: glycine cleavage system H protein, mitochondrial | XP_002280707.1 | *Vitis vinifera* | 26.98 | 5.77 | 20.85 | 4.07 | 99 | 18%(2) | 0.145 |
| E11 | Hypothetical protein | WP_039311068 | *Paenibacillus sp. IHB B 3415* | 28.98 | 7.75 | 22.31 | 4.25 | 239 | 15%(2) | 0.218 |
| E12 | PREDICTED: translationally-controlled tumor protein homolog | XP_008223198.1 | *Prunus mume* | 24.83 | 4.80 | 29.62 | 4.125 | 191 | 21%(3) | 0.173 |
| E13 | Hypothetical protein PRUPE_ppa009639mg | XP_007222184.1 | *Prunus persica* | 28.41 | 4.67 | 30.78 | 4.09 | 253 | 19%(3) | 0.037 |
| E14 | PREDICTED: uncharacterized protein At5g48480-like | XP_016484563.1 | *Nicotiana tabacum* | 27.37 | 5.07 | 28.12 | 4.40 | 258 | 27%(2) | 0.167 |
| E15 | Hypothetical protein CICLE_v10012331mg | XP_006428769.1 | *Citrus clementina* | 24.56 | 10.49 | 35.87 | 4.32 | 207 | 25%(3) | 0.147 |
| E16 | Chloroplast ribulose oxygenase | ABW80752 | *Flaveria bidentis* | 48.81 | 6.10 | 39.96 | 4.35 | 465 | 15%(5) | 0.304 |
| E17 | PREDICTED: uncharacterized protein At5g39570 | XP_002283932.1 | *Vitis vinifera* | 23.23 | 4.63 | 88.18 | 4.20 | 45 | 8%(1) | 0.382 |
| E18 | Ribulose-1,5-bisphosphate carboxylase/oxygenase large subunit, partial | ABB03930.1 | *Serpocaulon vacillans* | 9.92 | 9.66 | 74.70 | 6.7 | 71 | 12%(1) | 0.243 |
| E19 | Hypothetical protein VIGAN_06209900 | BAT90812.1 | *Vigna angularis var. angularis* | 31.86 | 6.90 | 43.70 | 8.27 | 330 | 13%(2) | 0.049 |
| E20 | Metal tolerance protein 4 (LOC18599142) | XM007028980.2 | *Theobroma cacao* | 39.45 | 5.82 | 43.96 | 6.14 | 409 | 53%(3) | 0.279 |

^a^ Accession number in NCBI database

^b^ Score is a measure of the statistical signiﬁcance of a match

^c^ Percentage of predicted protein sequence covered by matched peptides

^d^ Ratio means the ratio of Mn excess to adequate.

| **Primers** | **Sequence(5´-3´)** | **Remarks** |
| --- | --- | --- |
| CsMTP8-DEG-F | TCTANGGTTTCWAGTGCTGAYCCA | Degenerate primers |
| CsMTP8-DEG-R | AKGTCTACCTCRACAAAATAVAGA |
| CsMTP8-5’RACE-1 | CCTTAGGACCAAGTATGTCAGTTT | 5’ RACE |
| CsMTP8-5’RACE-2 | CTGTCAGCATAATGGCATACAACC |
| CsMTP8-3’RACE-1 | TCCTGTCGGTGCCATTGTCCTTGC | 3’ RACE |
| CsMTP8-3’RACE-2 | CACTGTCCGAGCCTACACTTTTGG |
| CsMTP8-FL-F | ATGGAAATGGGGATAGAAGCTCC | Amplification of *CsMTP8* open reading frame |
| CsMTP8-FL-R | AGGTTGGGTATTGGGGAGTCTGCT |
| CsMTP8-166-F | GCTCTAGAATGGAAATGGGGATAGAAGCTCC | Construction of pGFP-CsMTP8 fusion vector |
| CsMTP8-166-R | CGGGATCCAGGTTGGGTATTGGGGAGTCTGC |
| CsMTP8-2300-F | CGGGATCCATGGAAATGGGGATAGAAGCTCC | *CsMTP8-*overexpressing in *Arabidopsis* |
| CsMTP8-2300-R | GCTCTAGAAGGTTGGGTATTGGGGAGTCTGCT |
| CsMTP8-RT-F | TCTGGTTGGACAATCAGCTC | qRT-PCR of *CsMTP8* |
| CsMTP8-RT-R | TAGGCTCGGACAGTGTCAAC |
| Csβ-actin-F | GCCATCTTTGATTGGAATGG | Internal reference gene of *Camellia sinensis* |
| Csβ-actin-R | GGTGCCACAACCTTGATCTT |
| CsMTP8-pYES2-F | CGGGATCCAAAAAAATGGAAATGGGGATAGA | yeast transformation |
|
| CsMTP8-pYES2-R | GAATTCAGGTTGGGTATTGGGGAGTC |

Table S2. Oligonucleotides used in this study


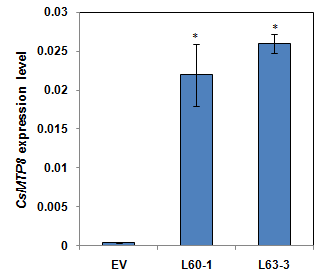


Figure S1. Expression analysis of CsMTP8 in transgenic Arabidopsis. Expression value was calculated based on comparison with *beta*-actin. Asterisks indicate a significant difference at the P = 0.05 level.


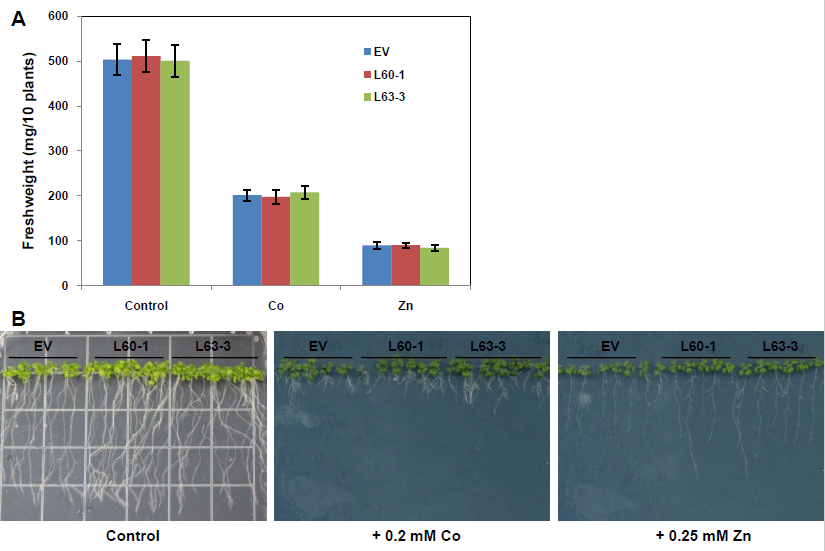


Figure S2. Effect of *CsMTP8* overexpression in *Arabidopsis thaliana* on Zn tolerance and Co. (A) Fresh weight of *A. thaliana* plants. (B) Growth of *A. thaliana* at different metal treatment for 14 days. 5-day-old seedlings were transferred into 1/2 MS supplemented with 2 mM or 4 mM for 12 days. EV indicates the wild-type Columbia ecotype of *A. thaliana* transformed with empty vector. L60-1, L63-3 are two transgenic lines expressing *CsMTP8*.
